# Supplementary material for: Elevated T cell repertoire diversity is associated with progression of lung squamous cell premalignant lesions
Source: J Immunother Cancer. 2021 Sep 27;9(9):e002647. doi: 10.1136/jitc-2021-002647 (PMC8477334; doi:10.1136/jitc-2021-002647)
Supplement: Supplementary data [file jitc-2021-002647supp001.pdf]

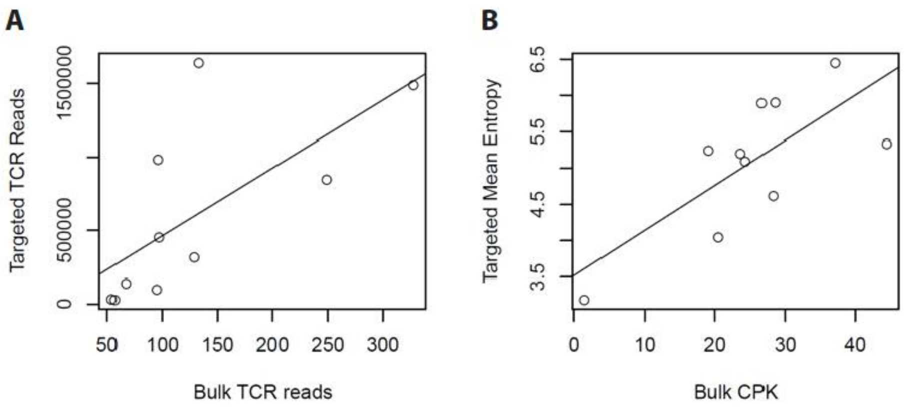

Supplementary Figure 1. TCR diversity is correlated between metrics derived from bulk RNA sequencing data and targeted TCR data. (A.) The number of total TCR reads correlated significantly between targeted (y-axis) and bulk RNA sequencing (x-axis) (rho = 0.70, p = 0.03). (**B**.) The clonotypes per kiloread (CPK) metric derived from the bulk RNA sequencing data (x-axis) is significantly correlated with the targeted TCR-seq metric of entropy (y-axis) (rho = 0.68, p = 0.029).

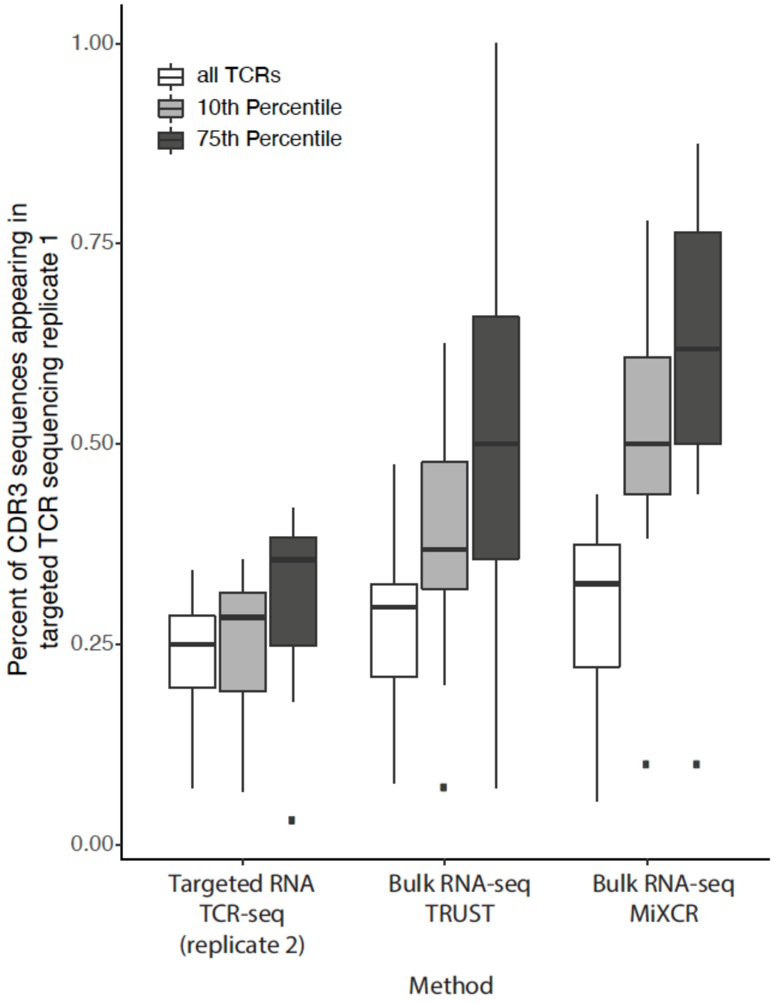

Supplementary Figure 2. Shared CDR3 amino acid sequences from bulk RNA-seq assembled TCRs and targeted RNA TCR-seq TCRs. The targeted RNA TCR sequencing generated data for 2 replicates per sample (n=11 samples). The boxplot shows the percent of CDR3 amino acid sequences from each method (Targeted RNA TCR-seq Replicate 2, Bulk RNA-seq TRUST, or Bulk RNA-seq MiXCR) that was also found in the targeted TCR-seq Replicate 1. Across all TCRs, no difference is found in the percent of CDR3 sequences from each group that appear in the replicate (Kruskal Wallace p>0.1). Percentages of shared CDR3 sequences were plotted for all TCR sequences (white boxes) or TCR sequences in at least the 10th (gray boxes) or 75th (dark gray boxes) percentiles of abundance for each sample in each method. When applying increasing thresholds of TCR abundance, all methods showed increased agreement with the TCR-seq replicate.

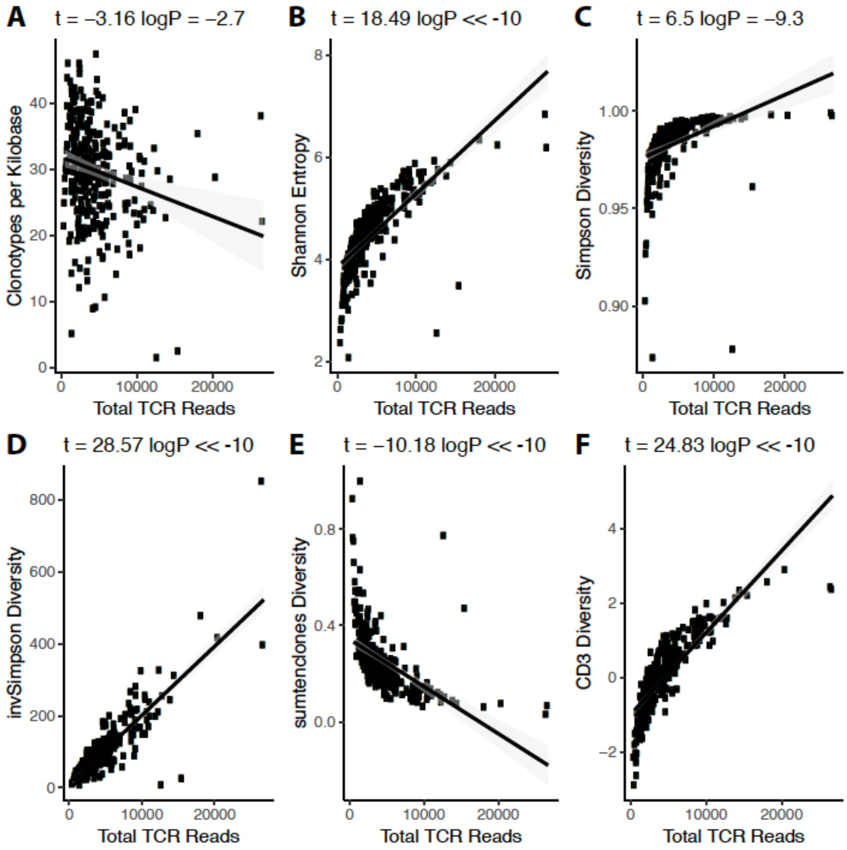

Supplementary Figure 3. Diversity metrics correlated with total number of TCR reads. The plots show the correlation between total TCR reads and diversity metrics and T cell gene expression. The plots are as follows: **(A.)** clonotypes per kiloread (CPK), **(B.)** Shannon entropy, **(C.)** Simpson diversity, **(D.)** inverse Simpson diversity, **(E.)** log10 sum of the top 10 clones, and **(F.)** CD3 gene expression. Clonotypes per kiloread (CPK) was the diversity metric that demonstrated the weakest correlation to total TCR reads. The correlation between metrics was derived using a linear mixed effects model adjusting for patient as a random effect and the t-statistic and log10 p-value are listed for each graph.

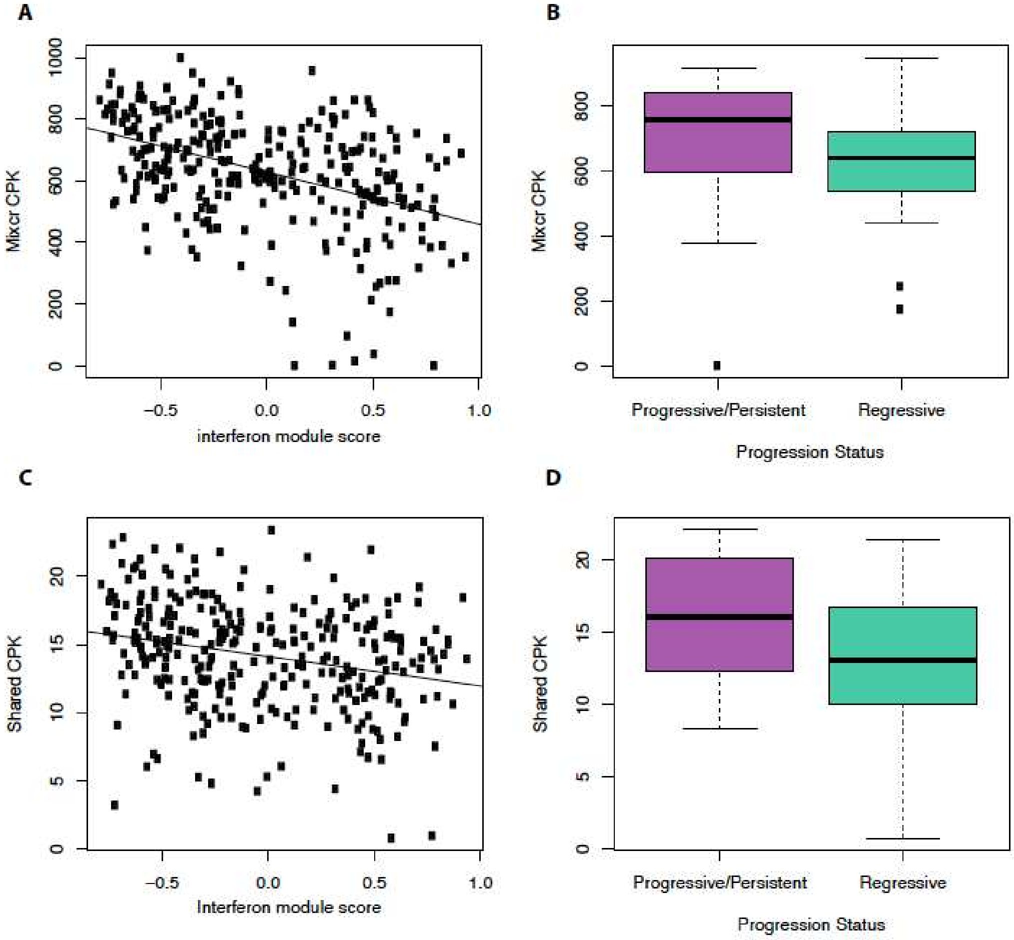

Supplementary Figure 4. TCR clonality is associated with immune processes and premalignant lesion outcome regardless of method used to assemble TCR sequences. We used the MiXCR sequences **(A.+B.)** or the shared TCR sequences found by MiXCR and TRUST **(C.+D.)** to compute Clonotypes per Kiloread (CPK) and correlate this metric with immune processes and premalignant lesion outcome. (**A.+ C.)** Clonotypes per Kiloread (CPK) is negatively correlated with a gene expression module of interferon response previously shown to be negatively associated with lesion progression: **(A.)** p = 7.6e-15 and **(D.)** p=2.2e-6. (**B.+D.)** Clonotypes per kiloread (CPK) in *Proliferative* lesions, by progression status (progressive/persistent lesions, purple; regressive lesions, turquoise). Progressive lesions have significantly higher CPK than regressing lesions (n=22 progressive/persistent and n=28 regressive samples, n=23 patients): **(B.)** p=0.04 and **(D.)** p=0.025.

BMJ Publishing Group Limited (BMJ) disclaims all liability and responsibility arising from any reliance placed on this supplemental material which has been supplied by the author(s)

PCGA ID	Chain	Total Reads	Unique CDR3 Reads
PCGA-01-0001-037-19118-00482BX-1007555R	TRA	35743	230
PCGA-01-0001-037-19118-00482BX-1007555R	TRA	33153	234
PCGA-01-0001-037-19118-00482BX-1007555R	TRB	12548	119
PCGA-01-0001-037-19118-00482BX-1007555R	TRB	11306	123
PCGA-01-0001-037-19118-00482BX-1007555R	TRD	734	10
PCGA-01-0001-037-19118-00482BX-1007555R	TRD	394	4
PCGA-01-0001-037-19118-00482BX-1007555R	TRG	3642	28
PCGA-01-0001-037-19118-00482BX-1007555R	TRG	2149	24
PCGA-01-0001-037-19762-00898BX-1007486R	TRA	470869	740
PCGA-01-0001-037-19762-00898BX-1007486R	TRA	461677	746
PCGA-01-0001-037-19762-00898BX-1007486R	TRB	147864	389
PCGA-01-0001-037-19762-00898BX-1007486R	TRB	170537	444
PCGA-01-0001-037-19762-00898BX-1007486R	TRD	31061	92
PCGA-01-0001-037-19762-00898BX-1007486R	TRD	40305	97
PCGA-01-0001-037-19762-00898BX-1007486R	TRG	81779	112
PCGA-01-0001-037-19762-00898BX-1007486R	TRG	85331	152
PCGA-01-0001-037-20126-01120BX-1031613R	TRA	124142	446
PCGA-01-0001-037-20126-01120BX-1031613R	TRA	110040	421
PCGA-01-0001-037-20126-01120BX-1031613R	TRB	69057	362
PCGA-01-0001-037-20126-01120BX-1031613R	TRB	58152	348
PCGA-01-0001-037-20126-01120BX-1031613R	TRD	7978	44
PCGA-01-0001-037-20126-01120BX-1031613R	TRD	7768	54
PCGA-01-0001-037-20126-01120BX-1031613R	TRG	11734	65
PCGA-01-0001-037-20126-01120BX-1031613R	TRG	11553	65
PCGA-01-0001-070-19300-00587BX-1007583R	TRA	125474	244
PCGA-01-0001-070-19300-00587BX-1007583R	TRA	136958	239
PCGA-01-0001-070-19300-00587BX-1007583R	TRB	66573	209
PCGA-01-0001-070-19300-00587BX-1007583R	TRB	40470	117
PCGA-01-0001-070-19300-00587BX-1007583R	TRD	21009	56
PCGA-01-0001-070-19300-00587BX-1007583R	TRD	12532	50
PCGA-01-0001-070-19300-00587BX-1007583R	TRG	22739	66
PCGA-01-0001-070-19300-00587BX-1007583R	TRG	28001	39
PCGA-01-0001-089-20126-01118BX-1031611R	TRA	263802	486
PCGA-01-0001-089-20126-01118BX-1031611R	TRA	245170	538
PCGA-01-0001-089-20126-01118BX-1031611R	TRB	119886	433
PCGA-01-0001-089-20126-01118BX-1031611R	TRB	90949	361
PCGA-01-0001-089-20126-01118BX-1031611R	TRD	30969	76
PCGA-01-0001-089-20126-01118BX-1031611R	TRD	39131	109
PCGA-01-0001-089-20126-01118BX-1031611R	TRG	21206	64
PCGA-01-0001-089-20126-01118BX-1031611R	TRG	39372	95
PCGA-01-0021-025-20449-01091BX-1031600R	TRA	7903	114
PCGA-01-0021-025-20449-01091BX-1031600R	TRA	8879	131
PCGA-01-0021-025-20449-01091BX-1031600R	TRB	5379	89
PCGA-01-0021-025-20449-01091BX-1031600R	TRB	5127	85
PCGA-01-0021-025-20449-01091BX-1031600R	TRD	178	3
PCGA-01-0021-025-20449-01091BX-1031600R	TRD	73	2
PCGA-01-0021-025-20449-01091BX-1031600R	TRG	648	17
PCGA-01-0021-025-20449-01091BX-1031600R	TRG	889	21
PCGA-01-0021-050-19434-00465BX-1007550R	TRA	103665	467
PCGA-01-0021-050-19434-00465BX-1007550R	TRA	97156	523
PCGA-01-0021-050-19434-00465BX-1007550R	TRB	48015	265
PCGA-01-0021-050-19434-00465BX-1007550R	TRB	40966	268
PCGA-01-0021-050-19434-00465BX-1007550R	TRD	4168	37
PCGA-01-0021-050-19434-00465BX-1007550R	TRD	1700	17
PCGA-01-0021-050-19434-00465BX-1007550R	TRG	15009	77
PCGA-01-0021-050-19434-00465BX-1007550R	TRG	8311	62
PCGA-01-0021-070-20449-01089BX-1031598R	TRA	361340	505
PCGA-01-0021-070-20449-01089BX-1031598R	TRA	569838	595
PCGA-01-0021-070-20449-01089BX-1031598R	TRB	304895	630
PCGA-01-0021-070-20449-01089BX-1031598R	TRB	332628	474
PCGA-01-0021-070-20449-01089BX-1031598R	TRD	10354	28
PCGA-01-0021-070-20449-01089BX-1031598R	TRD	28781	37
PCGA-01-0021-070-20449-01089BX-1031598R	TRG	25702	48
PCGA-01-0021-070-20449-01089BX-1031598R	TRG	8122	25
PCGA-01-0042-010-23911-01028BX-1033960R	TRA	217964	309
PCGA-01-0042-010-23911-01028BX-1033960R	TRA	304970	543
PCGA-01-0042-010-23911-01028BX-1033960R	TRB	219443	599
PCGA-01-0042-010-23911-01028BX-1033960R	TRB	174843	625
PCGA-01-0042-010-23911-01028BX-1033960R	TRD	1297	12
PCGA-01-0042-010-23911-01028BX-1033960R	TRG	32978	101
PCGA-01-0042-010-23911-01028BX-1033960R	TRG	31082	85
PCGA-01-0042-048-23680-00907BX-1033955R	TRA	9401	277
PCGA-01-0042-048-23680-00907BX-1033955R	TRA	9178	290
PCGA-01-0042-048-23680-00907BX-1033955R	TRB	7906	322
PCGA-01-0042-048-23680-00907BX-1033955R	TRB	6621	330
PCGA-01-0042-048-23680-00907BX-1033955R	TRD	15	3
PCGA-01-0042-048-23680-00907BX-1033955R	TRD	34	8
PCGA-01-0042-048-23680-00907BX-1033955R	TRG	877	32
PCGA-01-0042-048-23680-00907BX-1033955R	TRG	760	23
PCGA-01-0042-048-23911-01029BX-1034033R	TRA	39007	237
PCGA-01-0042-048-23911-01029BX-1034033R	TRA	42164	293
PCGA-01-0042-048-23911-01029BX-1034033R	TRB	27234	259
PCGA-01-0042-048-23911-01029BX-1034033R	TRB	24916	302
PCGA-01-0042-048-23911-01029BX-1034033R	TRD	92	3
PCGA-01-0042-048-23911-01029BX-1034033R	TRD	106	1
PCGA-01-0042-048-23911-01029BX-1034033R	TRG	3262	38
PCGA-01-0042-048-23911-01029BX-1034033R	TRG	3848	53

Supplementary Table 1. Samples that underwent targeted RNA TCR sequencing. For each sample, the chain sequenced, the total number of reads sequenced, and the number of unique CDR3 reads are listed.

	Expanded Clones									
**ID**	CASSPLKDGELFF	CQQYHDWSLYTF	CASSLAPPGSNQPQHF	CASSLAGSYNEQFF	CASSLAGTGEAFF	CASSQLTGYTGELFF	CASSLGSYEQYF	CASSSGQWYSYEQYF	CALWEPIYKKLF	CASSVEEGGYGYTF
PCGA-01-0001-037-19300-00589BX-1007585R	FALSE	FALSE	FALSE	FALSE	TRUE	FALSE	FALSE	FALSE	FALSE	FALSE
PCGA-01-0001-037-19762-00898BX-1007486R	FALSE	FALSE	FALSE	FALSE	TRUE	FALSE	FALSE	FALSE	FALSE	FALSE
PCGA-01-0001-076-20343-01314BX-1031646R	FALSE	FALSE	FALSE	FALSE	TRUE	FALSE	FALSE	FALSE	FALSE	FALSE
PCGA-01-0001-077-20126-01115BX-1031608R	FALSE	FALSE	FALSE	FALSE	TRUE	FALSE	FALSE	FALSE	FALSE	FALSE
PCGA-01-0012-050-21836-01253BX-1031633R	FALSE	FALSE	FALSE	FALSE	FALSE	FALSE	TRUE	FALSE	FALSE	FALSE
PCGA-01-0015-011-16541-00571BX-1007578R	FALSE	FALSE	FALSE	FALSE	FALSE	TRUE	FALSE	FALSE	FALSE	FALSE
PCGA-01-0015-011-17129-00932BX-1031579R	FALSE	FALSE	FALSE	FALSE	FALSE	TRUE	FALSE	FALSE	FALSE	FALSE
PCGA-01-0015-025-16954-00864BX-1007478R	FALSE	FALSE	FALSE	FALSE	FALSE	TRUE	FALSE	FALSE	FALSE	FALSE
PCGA-01-0029-050-22090-00380BX-1007537R	FALSE	FALSE	FALSE	FALSE	FALSE	FALSE	TRUE	TRUE	TRUE	TRUE
PCGA-01-0029-050-22678-00816BX-1007451R	FALSE	FALSE	FALSE	FALSE	FALSE	FALSE	TRUE	TRUE	TRUE	TRUE
PCGA-01-0029-061-22090-00378BX-1007535R	FALSE	FALSE	FALSE	FALSE	FALSE	FALSE	TRUE	FALSE	FALSE	FALSE
PCGA-01-0029-089-22090-00377BX-1007534R	FALSE	FALSE	FALSE	FALSE	FALSE	FALSE	FALSE	FALSE	FALSE	TRUE
PCGA-01-0031-053-24726-01061BX-1031594R	FALSE	FALSE	FALSE	TRUE	FALSE	FALSE	FALSE	FALSE	FALSE	FALSE
PCGA-01-0031-074-24285-00854BX-1007472R	FALSE	FALSE	TRUE	TRUE	FALSE	FALSE	FALSE	FALSE	FALSE	FALSE
PCGA-01-0031-074-24726-01060BX-1031593R	FALSE	FALSE	TRUE	TRUE	FALSE	FALSE	FALSE	FALSE	FALSE	FALSE
PCGA-01-0044-048-17646-44967BX-1033975R	FALSE	TRUE	FALSE	FALSE	FALSE	FALSE	FALSE	FALSE	FALSE	FALSE
PCGA-01-0044-048-17898-01132BX-1034080R	FALSE	TRUE	FALSE	FALSE	FALSE	FALSE	FALSE	FALSE	FALSE	FALSE
PCGA-01-0044-050-17898-01133BX-1034071R	TRUE	FALSE	FALSE	FALSE	FALSE	FALSE	FALSE	FALSE	FALSE	FALSE
PCGA-01-0044-070-17646-44965BX-1033993R	TRUE	FALSE	FALSE	FALSE	FALSE	FALSE	FALSE	FALSE	FALSE	FALSE
PCGA-01-0044-070-17898-01130BX-1034098R	FALSE	TRUE	FALSE	FALSE	FALSE	FALSE	FALSE	FALSE	FALSE	FALSE
PCGA-01-0044-089-17646-44966BX-1033984R	TRUE	FALSE	FALSE	FALSE	FALSE	FALSE	FALSE	FALSE	FALSE	FALSE
PCGA-01-0044-089-17898-01131BX-1034089R	TRUE	FALSE	FALSE	FALSE	FALSE	FALSE	FALSE	FALSE	FALSE	FALSE
Public/Private TCR	Private	Private	Private	Private	Private	Private	Public	Private	Private	Private
Number Time Points	2	2	2	2	4	3	3	2	2	2
Number of Locations	3	2	1	1	3	2	2	1	1	2
Number of Time Points/Locations	2	2	2	2	2	2	2	2	2	2
Number of Patients	1	1	1	1	1	1	2	1	1	1

Supplementary Table 2. Expanded TCR clones at multiple time points within the same lesion location. For each expanded clone (columns), the samples (rows) are marked as TRUE if the clone was present or FALSE if it was absent. The clones are classified as private (found only in the sample from a single patient) versus public (found in multiple patients) and summarized by the number of time points, locations, or unique locations across time that each expanded clone is present in.

	Sample Characteristics												
ID	Patient	Lung Location Code	Lung Location Name	Lung Location Description	Time of Sample Collection	Histology	Histology Code	Complete Previous Histology at Site	Complete Later Histology at Site	Molecular Subtype	Progression/Regression Status	Genomic Smoking Status	Previous History of Lung Cancer
PCGA-01-0001-037-19300-00589BX-1007585R	1	37	LGLO	Lingula Orifice or opening	19300	Normal	4	25	1,22.3,22.3	Secretory	Normal/Stable	Current	N
PCGA-01-0001-037-19762-00898BX-1007486R	1	37	LGLO	Lingula Orifice or opening	19762	Normal	1	25,4	22.3,22.3	Secretory	Normal/Stable	Current	N
PCGA-01-0001-076-20343-01314BX-1031646R	1	76	RB8	RLL Anterior Basal Seg (ABS)	20343	Normal	4	25,24		Normal	UNK	Current	N
PCGA-01-0001-077-20126-01115BX-1031608R	1	77	RB8/9	RLL ABS Carina between RB8 and RB9	20126	Hyperplasia	22.3	1		Secretory	UNK	Current	N
PCGA-01-0012-050-21836-01253BX-1031633R	12	50	MC	Main Carina, Carina NOS	21836	Hyperplasia	22.3	22,5	22.3	Inflammatory	Normal/Stable	Former	Y
PCGA-01-0015-011-16541-00571BX-1007578R	15	11	LB1/2	LUL APS Carina between LB1 and LB2	16541	ModD	25	25	24,26	Secretory	Progressive/Persistent	Current	Y
PCGA-01-0015-011-17129-00932BX-1031579R	15	11	LB1/2	LUL APS Carina between LB1 and LB2	17129	MildD	24	25,25	26	Inflammatory	Progressive/Persistent	Former	Y
PCGA-01-0015-025-16954-00864BX-1007478R	15	25	LB6	LLL Superior Segment (SS)	16954	Metaplasia	23	25,24	24	Inflammatory	Progressive/Persistent	Current	Y
PCGA-01-0029-050-22090-00380BX-1007537R	29	50	MC	Main Carina, Carina NOS	22090	ModD	25	22.3,24	NA,23	Proliferative	Regressive	Current	Y
PCGA-01-0029-050-22678-00816BX-1007451R	29	50	MC	Main Carina, Carina NOS	22678	Metaplasia	23	22.3,24,25,NA		Secretory	UNK	Current	Y
PCGA-01-0029-061-22090-00378BX-1007535R	29	61	RB2/3	RUL Carina between RB2 and RB3	22090	ModD	25	1.5,26	25,24	Secretory	Progressive/Persistent	Current	Y
PCGA-01-0029-089-22090-00377BX-1007534R	29	89	RMLO	Right Middle Lobe Orifice or opening	22090	MildD	24	23,26,26	23	Secretory	Regressive	Current	Y
PCGA-01-0031-053-24726-01061BX-1031594R	31	53	RB1/2	RUL Carina between RB1 and RB2	24726	Hyperplasia	22.4	25,23	22.3,0	Proliferative	Normal/Stable	Current	N
PCGA-01-0031-074-24285-00854BX-1007472R	31	74	RB7	RLL Medial Basal Segment (MBS)	24285	Hyperplasia	22.3		22.4,22.4	Inflammatory	Normal/Stable	Current	N
PCGA-01-0031-074-24726-01060BX-1031593R	31	74	RB7	RLL Medial Basal Segment (MBS)	24726	Hyperplasia	22.4	22.3	22.4	Inflammatory	Normal/Stable	Current	N
PCGA-01-0044-048-17646-44967BX-1033975R	44	48	LULO	Left Upper Lobe Orifice or opening	17646	Hyperplasia	22.3		4	Inflammatory	Normal/Stable	Former	N
PCGA-01-0044-048-17898-01132BX-1034080R	44	48	LULO	Left Upper Lobe Orifice or opening	17898	Normal	4	22.3		Inflammatory	UNK	Former	N
PCGA-01-0044-050-17898-01133BX-1034071R	44	50	MC	Main Carina, Carina NOS	17898	ModD	25	23,25	23	Proliferative	Regressive	Former	N
PCGA-01-0044-070-17646-44965BX-1033993R	44	70	RB6	RLL Superior Basal Segment (SBS)	17646	Hyperplasia	22.3	4	22.3,4	Secretory	Normal/Stable	Former	N
PCGA-01-0044-070-17898-01130BX-1034098R	44	70	RB6	RLL Superior Basal Segment (SBS)	17898	Hyperplasia	22.3	4,22.3	4	Inflammatory	Normal/Stable	Former	N
PCGA-01-0044-089-17646-44966BX-1033984R	44	89	RMLO	Right Middle Lobe Orifice or opening	17646	Hyperplasia	22.3		22.3,22.3	Secretory	Normal/Stable	Former	N
PCGA-01-0044-089-17898-01131BX-1034089R	44	89	RMLO	Right Middle Lobe Orifice or opening	17898	Hyperplasia	22.3	22.3	22.3	Secretory	Normal/Stable	Former	N

Supplementary Table 3. Phenotypic data across the samples where expanded TCR clones are present. For each sample ID the following characteristics are listed: Patient ID, location code for the sample, location name, location description, time of collection, histology (MildD = mild dysplasia, ModD = moderate dysplasia, SevD = severe dysplasia), histology code, histology code(s) for lesions sampled in the past from that location, histology code(s) for lesions sampled in the future from that location, molecular subtype, progression/regression status, genomic smoking status, and prior history of lung cancer. Histology codes are as follow: normal (<22),

hyperplasia (>=22 and <23), metaplasia (23), mild dysplasia (24), moderate dysplasia (25), severe dysplasia (26), and unknown histology/insufficient material (NA).
